# Supplementary material for: Metabolomic Profiling in Combination with Data Association Analysis Provide Insights about Potential Metabolic Regulation Networks among Non-Volatile and Volatile Metabolites in Camellia sinensis cv Baijiguan
Source: Plants (Basel). 2022 Sep 28;11(19):2557. doi: 10.3390/plants11192557 (PMC9572950; doi:10.3390/plants11192557)
Supplement: Supplementary file 1 [file plants-11-02557-s001.zip › plants-1866919-supplementary/Supplementary material/Supplementary Data S1.pdf]

Supplementary Data S1. The nonvolatile contents of the albino half-sibs and the green half-sibs of *Camellia sinensis cv Baijiguan* (%).

	Germplasm	Total polyphenols	Amino Acids	Caffeine	GA	GC	EGC	C	EC	EGCG	GCG	ECG	CG	Non-ester catechins	Ester-catechins	Total catechins
Albino half-sibs	0306C	20.10±1.60	5.35±0.37	3.35±0.17	0.00±0.00	0.00±0.00	1.54±0.52	0.30±0.08	0.80±0.05	5.16±1.26	1.31±0.11	1.82±0.45	0.00±0.00	2.54±0.46	7.41±1.33	10.64±1.59
	0306D	17.90±1.83	4.67±0.59	3.27±0.15	0.05±0.01	0.43±0.00	1.32±0.32	0.15±0.03	0.58±0.13	6.44±0.97	1.11±0.01	1.61±0.33	0.33±0.01	2.27±0.31	8.77±0.95	11.05±0.95
	0306F	16.99±1.14	6.51±0.10	3.81±0.01	0.06±0.01	0.16±0.01	1.51±0.29	0.20±0.04	1.09±0.24	6.74±0.56	0.10±0.01	3.15±0.67	0.00±0.00	2.85±0.11	9.92±1.07	12.79±1.16
	0306I	20.47±2.03	6.22±0.62	3.90±0.13	0.00±0.00	0.00±0.00	0.80±0.28	0.33±0.09	0.59±0.05	6.09±1.01	1.49±0.11	1.90±0.32	0.00±0.00	1.61±0.27	8.48±0.89	9.90±1.59
	0317L	20.56±0.80	6.26±0.19	3.47±0.23	0.00±0.00	0.00±0.00	1.06±0.41	0.24±0.03	0.49±0.04	6.92±0.78	2.19±0.20	1.83±0.13	0.00±0.00	1.71±0.35	9.48±0.35	10.95±0.60
	0317N	15.95±0.45	7.36±0.18	3.43±0.25	0.00±0.00	0.11±0.02	1.33±0.24	0.36±0.06	0.86±0.26	5.72±0.95	0.79±0.54	1.95±0.49	0.00±0.00	2.46±0.24	8.2±1.06	10.94±1.80
Green half-sibs	0306A	21.32±1.37	5.96±0.43	3.46±0.10	0.09±0.01	0.15±0.01	1.85±0.57	0.31±0.08	1.35±0.30	7.85±1.41	0.90±0.60	2.72±0.48	0.00±0.00	3.57±0.79	11.17±1.35	14.74±2.14
	0306B	20.72±0.23	3.27±0.40	3.53±0.10	0.00±0.00	0.33±0.01	1.39±0.21	0.18±0.01	0.76±0.02	7.04±0.62	0.80±0.02	2.83±0.07	0.09±0.00	2.32±0.06	10.17±0.03	11.65±0.08
	0306H	19.98±1.62	5.70±0.39	3.32±0.04	0.00±0.00	0.37±0.04	1.61±0.23	0.31±0.08	1.16±0.50	7.84±0.86	0.09±0.01	2.47±0.67	0.00±0.00	3.09±0.92	10.34±1.56	15.16±2.78
	0306L	24.28±1.19	6.84±0.50	3.25±0.08	0.00±0.00	0.41±0.01	1.28±0.16	0.15±0.07	0.62±0.07	7.30±1.05	1.13±0.29	1.65±0.18	0.30±0.03	2.26±0.11	9.95±0.71	12.20±0.93
	0309A	21.40±1.76	4.97±0.16	3.00±0.05	0.08±0.01	0.00±0.00	2.00±0.36	0.28±0.07	0.92±0.11	8.16±0.70	0.00±0.00	2.68±0.07	0.00±0.00	3.2±0.40	10.84±0.76	14.04±1.13
	0317D	22.47±1.32	4.97±0.13	3.07±0.18	0.05±0.01	0.29±0.02	1.77±0.19	0.20±0.04	0.80±0.06	7.88±0.58	0.70±0.02	1.97±0.21	0.16±0.01	2.91±0.18	10.28±0.45	13.19±0.67

Data were presented as average ± standard error (n=4). GA: gallic acid; GC: (+)-gallocatechin; EGC: (-)-epigallocatechin; C: (+)-catechin; EC: (-)-epicatechin; EGCG: (-)-epigallocatechin gallate; GCG: gallocatechin gallate; ECG: (-)-epicatechin gallate; CG: catechin gallate.
