# Supplementary material for: Metabolomic Profiling in Combination with Data Association Analysis Provide Insights about Potential Metabolic Regulation Networks among Non-Volatile and Volatile Metabolites in Camellia sinensis cv Baijiguan
Source: Plants (Basel). 2022 Sep 28;11(19):2557. doi: 10.3390/plants11192557 (PMC9572950; doi:10.3390/plants11192557)
Supplement: Supplementary file 1 [file plants-11-02557-s001.zip › plants-1866919-supplementary/Supplementary material/Supplementary Data S2.pdf]

Supplementary Data S2. The volatile contents (μg g^{-1} fresh tea weight) from the albino half-sibs and the green half-sibs of *Camellia sinensis cv Baijiguan*

Pathway	RT (min)	Compound	Contents (μg g^{-1} fresh tea weight)											
			0306C	0306D	0306F	0306I	0317L	0317N	0306A	0306B	0306H	0306L	0309A	0317D
Phenylpropanoid	10.15	Benzyl alcohol	2.82±0.16	4.46±0.59	2.62±0.48	5.94±0.25	7.29±0.62	0.63±0.08	3.74±0.20	2.93±0.20	12.87±0.88	4.00±0.31	15.03±0.50	4.12±0.34
	12.57	Phenylethyl Alcohol	12.80±1.20	14.09±1.02	2.19±0.45	3.54±0.30	11.92±0.71	2.00±0.07	5.64±0.46	8.82±0.47	7.25±0.39	15.67±1.37	17.53±0.22	16.49±0.22
	15.04	Methyl salicylate	3.82±0.27	2.33±0.15	4.44±0.37	4.77±0.28	4.37±0.19	1.63±0.11	1.91±0.12	5.31±0.54	2.70±0.12	2.93±0.18	4.51±0.08	7.47±0.65
	18.84	Indole	0.08±0.00	0.17±0.00	0.15±0.02	0.16±0.02	0.14±0.01	0.08±0.00	0.12±0.00	0.21±0.01	0.16±0.01	0.11±0.00	0.17±0.01	0.11±0.02
	22.38	Coumarin	0.05±0.00	0.13±0.00	0.12±0.01	0.12±0.02	nd	nd	0.07±0.00	0.32±0.04	0.08±0.01	nd	nd	0.06±0.01
		subtotal	19.56±1.54	21.18±1.55	9.53±0.88	14.53±0.76	23.72±1.41	4.35±0.11	11.48±0.61	17.58±0.50	23.06±0.59	22.72±1.61	37.24±0.56	28.26±1.17
MEP	8.82	(E)-β-Ocimene	0.54±0.02	0.76±0.07	0.82±0.13	0.69±0.07	0.50±0.02	0.45±0.16	0.48±0.09	2.64±0.32	1.57±0.12	0.55±0.02	0.58±0.25	1.21±0.16
	9.61	α-Terpinene	0.00±0.00	0.00±0.00	0.00±0.00	0.00±0.00	0.00±0.00	0.15±0.03	0.00±0.00	0.00±0.00	0.00±0.00	0.00±0.00	0.09±0.01	0.00±0.00
	10.41	cis-Linalool oxide (furanoid)	2.79±0.31	3.65±0.08	5.77±1.49	5.93±0.35	3.39±0.28	1.30±0.09	2.72±0.17	1.01±0.51	3.09±0.25	2.26±0.24	3.77±0.47	4.92±0.50
	11.30	trans-Linalool oxide (furanoid)	2.08±0.12	1.23±0.17	1.85±0.17	1.79±0.06	1.74±0.09	0.20±0.02	1.59±0.10	0.97±0.07	2.37±0.18	0.91±0.07	0.96±0.06	5.62±0.18
	11.79	Linalool	3.84±0.16	2.08±0.22	3.79±0.34	3.99±0.16	3.13±0.12	0.28±0.03	3.53±0.24	1.80±0.02	5.17±0.46	1.73±0.12	2.14±0.07	11.66±0.64
	14.36	cis-Linalool oxide (pyranoid)	nd	nd	nd	nd	0.05±0.00	nd	nd	nd	0.09±0.01	nd	0.13±0.02	nd
	14.50	trans-Linalool oxide (pyranoid)	0.73±0.03	0.43±0.06	0.00±0.00	0.52±0.02	0.60±0.02	0.07±0.00	0.55±0.03	0.00±0.00	1.19±0.11	0.28±0.03	0.61±0.03	2.23±0.04
	16.76	Geraniol	1.60±0.17	0.33±0.07	0.00±0.00	0.38±0.04	1.28±0.09	0.22±0.03	0.83±0.08	1.34±0.24	4.55±0.23	1.00±0.15	5.73±0.73	4.26±0.45
	17.13	Nerol	0.34±0.02	0.09±0.01	0.16±0.02	0.23±0.02	0.17±0.04	0.11±0.02	0.28±0.05	0.65±0.09	0.11±0.01	0.20±0.03	0.06±0.00	0.64±0.09
	17.51	Neral	0.12±0.00	0.24±0.01	0.23±0.02	0.22±0.02	0.17±0.01	0.13±0.01	0.15±0.00	0.23±0.01	0.15±0.00	0.14±0.00	0.23±0.01	0.12±0.01
	34.92	Geranyllinalool	0.05±0.00	0.08±0.01	0.10±0.02	0.11±0.01	0.06±0.01	0.05±0.01	0.06±0.01	0.00±0.00	0.15±0.02	0.09±0.04	0.10±0.03	0.07±0.01

	30.88	Neophytadiene	0.09±0.05	0.13±0.06	0.49±0.31	0.15±0.05	0.14±0.08	0.11±0.03	0.06±0.01	0.14±0.05	0.09±0.05	0.08±0.03	0.17±0.04	0.26±0.10
		Subtotal	12.18±0.44	9.02±0.36	13.21±1.69	14.01±0.48	11.24±0.50	3.06±0.11	10.24±0.56	8.79±0.63	18.53±0.63	7.24±0.60	14.57±0.40	31.00±1.49
MVA	20.60	α-Copaene	0.04±0.00	0.06±0.00	0.00±0.00	0.04±0.01	0.09±0.01	0.03±0.00	0.05±0.00	0.39±0.06	0.06±0.00	0.04±0.00	0.06±0.01	0.13±0.01
	21.66	Caryophyllene	nd	nd	nd	nd	0.08±0.01	nd	0.04±0.01	0.26±0.04	0.04±0.00	nd	0.07±0.01	nd
	22.44	Humulene	0.29±0.02	0.32±0.04	0.00±0.00	0.34±0.01	0.35±0.01	0.27±0.02	0.33±0.01	0.69±0.06	0.07±0.00	0.32±0.01	0.39±0.02	0.10±0.02
	22.60	β-Farnesene	0.06±0.00	0.09±0.00	0.08±0.01	0.10±0.00	0.07±0.00	0.04±0.00	0.08±0.00	0.20±0.01	0.14±0.01	0.04±0.00	0.06±0.00	0.09±0.01
	23.68	Germacrene D	nd	0.04±0.00	0.07±0.01	0.04±0.00	0.04±0.00	0.03±0.00	nd	nd	nd	nd	0.04±0.00	0.08±0.01
	23.43	α-Muurolene	nd	0.04±0.00	0.05±0.01	nd	nd	nd	0.04±0.00	nd	0.06±0.00	nd	nd	nd
	24.25	α-Farnesene	0.16±0.00	0.26±0.01	0.29±0.03	0.28±0.02	0.24±0.02	0.17±0.00	0.23±0.01	0.39±0.00	0.35±0.01	0.17±0.01	0.19±0.01	0.19±0.02
	24.48	δ-Cadinene	0.03±0.00	0.04±0.01	0.05±0.01	0.03±0.00	0.06±0.01	nd	0.05±0.01	0.42±0.07	0.05±0.00	nd	0.04±0.00	0.11±0.01
	24.56	α-Cadinene	0.04±0.00	0.11±0.01	nd	nd	0.10±0.01	0.06±0.00	0.09±0.00	0.23±0.01	0.13±0.00	0.06±0.00	0.10±0.00	nd
	24.93	(E)-Nerolidol	0.00±0.00	0.07±0.01	0.08±0.01	0.07±0.00	0.10±0.01	0.03±0.00	0.06±0.00	0.11±0.01	0.06±0.01	0.07±0.00	0.08±0.01	0.08±0.00
	25.11	Caryophyllene oxide	nd	nd	0.06±0.00	0.06±0.00	nd	0.04±0.00	nd	0.10±0.01	0.07±0.01	nd	0.07±0.01	0.06±0.01
	25.55	Germacrene B	0.16±0.04	0.04±0.00	0.09±0.01	0.07±0.01	0.08±0.02	0.03±0.00	0.05±0.00	0.23±0.02	nd	0.11±0.02	0.53±0.12	nd
	26.49	Cubebol	nd	nd	nd	nd	0.08±0.00	0.06±0.00	nd	nd	0.09±0.01	0.08±0.00	0.09±0.01	nd
	27.05	α-Cadinol	0.12±0.07	0.19±0.08	0.81±0.39	0.23±0.05	nd	nd	0.10±0.03	0.16±0.04	nd	nd	nd	0.49±0.15
		Subtotal	0.91±0.08	1.27±0.12	1.57±0.40	1.26±0.08	1.28±0.04	0.75±0.02	1.14±0.05	3.19±0.26	1.11±0.04	0.89±0.01	1.71±0.14	1.33±0.18
FA-derivative	20.44	cis-3-hexenyl hexanoate	0.04±0.00	0.06±0.00	0.07±0.01	0.05±0.00	0.05±0.00	0.03±0.00	0.04±0.00	0.11±0.00	0.09±0.00	0.04±0.00	0.06±0.00	0.00±0.00
	21.05	cis-Jasmone	0.10±0.00	0.23±0.00	0.23±0.03	0.21±0.03	0.19±0.01	0.12±0.00	0.14±0.00	0.50±0.03	0.30±0.02	0.11±0.00	0.21±0.01	0.00±0.00
	33.41	Hexadecanoic acid, methyl ester	0.04±0.01	0.07±0.01	0.10±0.03	0.10±0.00	nd	nd	0.07±0.01	0.12±0.01	nd	nd	nd	0.06±0.00
		subtotal	0.19±0.01	0.36±0.02	0.40±0.05	0.36±0.04	0.23±0.01	0.15±0.01	0.26±0.01	0.72±0.05	0.39±0.02	0.15±0.00	0.27±0.02	0.06±0.00

Data were expressed as mean ± standard error (n = 4). nd, not detected.
