# Supplementary material for: Metabolomic Profiling in Combination with Data Association Analysis Provide Insights about Potential Metabolic Regulation Networks among Non-Volatile and Volatile Metabolites in Camellia sinensis cv Baijiguan
Source: Plants (Basel). 2022 Sep 28;11(19):2557. doi: 10.3390/plants11192557 (PMC9572950; doi:10.3390/plants11192557)
Supplement: Supplementary file 1 [file plants-11-02557-s001.zip › plants-1866919-supplementary/Supplementary material/Supplementary Data S3.pdf]

**Supplementary Data S3.** The volatile compositions (Mol%) from the albino half-sibs and the green half-sibs of *Camellia sinensis cv Baijiguan*

| Pathway | RT (min) | Compound | Contents ((Mol%) | | | | | | | | | | | |
|---|---|---|---|---|---|---|---|---|---|---|---|---|---|---|
| | | | 0306C | 0306D | 0306F | 0306I | 0317L | 0317N | 0306A | 0306B | 0306H | 0306L | 0309A | 0317D |
| Phenylpropanoid | 10.15 | Benzyl alcohol | 16.78±1.00 | 23.46±1.95 | 32.63±3.81 | 46.88±0.75 | 34.50±1.20 | 17.35±2.03 | 36.59±1.88 | 19.71±2.04 | 59.94±2.79 | 20.13±1.73 | 44.29±0.80 | 16.98±0.92 |
| | 12.57 | Phenylethyl Alcohol | 66.52±1.13 | 66.37±2.02 | 24.20±3.96 | 24.54±0.84 | 50.10±1.12 | 48.68±0.76 | 48.51±1.68 | 52.21±2.47 | 30.10±2.21 | 68.96±1.75 | 45.78±0.51 | 60.54±1.77 |
| | 15.04 | Methyl salicylate | 16.05±0.60 | 8.81±0.23 | 40.23±3.00 | 26.70±0.61 | 14.80±0.28 | 31.93±2.59 | 13.29±0.70 | 25.23±2.66 | 9.01±0.60 | 10.41±0.11 | 9.47±0.34 | 21.86±1.20 |
| | 18.84 | Indole | 0.45±0.04 | 0.85±0.06 | 1.82±0.26 | 1.16±0.19 | 0.60±0.02 | 2.04±0.09 | 1.09±0.07 | 1.29±0.02 | 0.68±0.02 | 0.49±0.04 | 0.46±0.04 | 0.43±0.09 |
| | 22.38 | Coumarin | 0.20±0.02 | 0.51±0.03 | 1.12±0.17 | 0.72±0.16 | nd | nd | 0.53±0.04 | 1.56±0.20 | 0.27±0.02 | nd | nd | 0.19±0.03 |
| | | subtotal | 100 | 100 | 100 | 100 | 100 | 100 | 100 | 100 | 100 | 100 | 100 | 100 |
| MEP | 8.82 | (E)-β-Ocimene | 5.24±0.27 | 10.15±1.17 | 7.60±0.88 | 5.93±0.68 | 5.30±0.07 | 16.82±5.27 | 5.63±1.02 | 33.83±3.70 | 9.97±1.01 | 9.15±0.73 | 4.52±1.96 | 4.62±0.69 |
| | 9.61 | α-Terpinene | 0.00±0.00 | 0.00±0.00 | 0.00±0.00 | 0.00±0.00 | 0.00±0.00 | 6.02±1.52 | 0.00±0.00 | 0.00±0.00 | 0.00±0.00 | 0.00±0.00 | 0.75±0.10 | 0.00±0.00 |
| | 10.41 | cis-Linalool oxide (furanoid) | 21.65±2.29 | 39.01±2.02 | 40.57±4.86 | 40.57±1.23 | 28.68±1.22 | 40.28±2.88 | 25.10±0.58 | 9.74±4.60 | 15.65±1.48 | 29.50±1.16 | 24.63±3.78 | 14.94±1.29 |
| | 11.30 | trans-Linalool oxide (furanoid) | 16.17±0.58 | 12.95±1.27 | 14.01±1.74 | 12.31±0.53 | 14.82±0.53 | 6.15±0.75 | 14.71±0.16 | 10.22±1.56 | 11.90±0.61 | 11.99±0.29 | 6.22±0.48 | 17.11±0.31 |
| | 11.79 | Linalool | 32.98±0.97 | 24.20±1.71 | 31.72±3.80 | 30.26±1.05 | 29.32±0.21 | 9.52±0.91 | 35.93±0.63 | 20.55±1.58 | 28.58±1.59 | 25.13±1.19 | 15.32±0.94 | 39.10±0.28 |
| | 14.36 | cis-Linalool oxide (pyranoid) | nd | nd | nd | nd | 0.43±0.02 | nd | nd | nd | 0.45±0.05 | nd | 0.87±0.14 | nd |
| | 14.50 | trans-Linalool oxide (pyranoid) | 5.68±0.20 | 4.55±0.47 | 0.00±0.00 | 3.59±0.13 | 5.07±0.07 | 2.32±0.14 | 5.10±0.29 | 0.00±0.00 | 5.94±0.34 | 3.63±0.08 | 4.00±0.29 | 6.83±0.36 |
| | 16.76 | Geraniol | 13.65±1.11 | 3.80±0.83 | 0.00±0.00 | 2.84±0.26 | 12.00±0.71 | 7.55±0.99 | 8.42±0.48 | 14.79±1.82 | 25.31±1.25 | 14.23±1.33 | 40.55±4.25 | 14.20±0.96 |

| | | | | | | | | | | | | | | |
|---|---|---|---|---|---|---|---|---|---|---|---|---|---|---|
| | 17.13 | Nerol | 2.94±0.13 | 1.10±0.17 | 1.41±0.31 | 1.77±0.19 | 1.69±0.39 | 3.80±0.68 | 2.93±0.51 | 7.30±0.65 | 0.63±0.09 | 2.94±0.29 | 0.45±0.05 | 2.13±0.20 |
| | 17.51 | Neral | 1.05±0.06 | 2.86±0.15 | 1.94±0.23 | 1.71±0.15 | 1.66±0.12 | 4.53±0.48 | 1.53±0.10 | 2.61±0.24 | 0.82±0.02 | 2.15±0.16 | 1.64±0.08 | 0.43±0.04 |
| | 34.92 | Geranyllinalool | 0.21±0.01 | 0.52±0.09 | 0.44±0.12 | 0.42±0.02 | 0.31±0.03 | 0.91±0.19 | 0.32±0.04 | 0.00±0.00 | 0.46±0.08 | 0.64±0.21 | 0.37±0.09 | 0.13±0.01 |
| | 30.88 | Neophytadiene | 0.44±0.21 | 0.86±0.39 | 2.32±1.62 | 0.61±0.19 | 0.73±0.45 | 2.10±0.63 | 0.34±0.07 | 0.95±0.38 | 0.29±0.16 | 0.63±0.22 | 0.69±0.14 | 0.51±0.21 |
| | | Subtotal | 100 | 100 | 100 | 100 | 100 | 100 | 100 | 100 | 100 | 100 | 100 | 100 |
| MVA | 20.60 | α-Copaene | 4.73±0.58 | 5.06±0.62 | 0.00±0.00 | 3.51±0.46 | 6.77±0.51 | 3.94±0.15 | 4.59±0.20 | 12.30±1.02 | 5.11±0.12 | 4.41±0.23 | 3.32±0.43 | 10.52±1.97 |
| | 21.66 | Caryophyllene | nd | nd | nd | nd | 6.02±0.51 | nd | 3.83±0.39 | 8.20±0.71 | 3.46±0.22 | nd | 4.07±0.61 | nd |
| | 22.44 | Humulene | 33.72±4.26 | 25.19±1.66 | 0.00±0.00 | 27.82±0.94 | 27.96±0.28 | 35.77±1.35 | 29.61±1.06 | 21.95±0.49 | 6.45±0.28 | 36.66±1.45 | 23.19±2.01 | 8.18±1.87 |
| | 22.60 | β-Farnesene | 6.59±0.70 | 7.53±0.79 | 6.38±1.03 | 7.82±0.28 | 5.22±0.24 | 5.25±0.16 | 7.22±0.54 | 6.40±0.14 | 12.41±0.70 | 4.24±0.23 | 3.70±0.14 | 7.07±0.98 |
| | 23.68 | Germacrene D | nd | 3.15±0.22 | 5.28±1.36 | 3.58±0.36 | 3.32±0.16 | 3.73±0.15 | nd | nd | nd | nd | 2.11±0.22 | 6.75±0.69 |
| | 23.43 | α-Muurolene | 0.00±0.00 | 3.37±0.31 | 3.77±0.97 | 0.00±0.00 | 0.00±0.00 | 0.00±0.00 | 3.70±0.17 | 0.00±0.00 | 5.78±0.23 | 0.00±0.00 | 0.00±0.00 | 0.00±0.00 |
| | 24.25 | α-Farnesene | 17.90±1.20 | 21.49±1.23 | 21.75±3.44 | 22.75±2.01 | 18.76±1.49 | 22.69±0.42 | 20.74±0.63 | 12.49±1.01 | 31.67±0.41 | 19.57±0.74 | 11.60±0.70 | 15.20±1.80 |
| | 24.48 | δ-Cadinene | 3.76±0.58 | 3.54±0.19 | 3.89±1.14 | 2.80±0.07 | 4.44±0.48 | 0.00±0.00 | 4.73±0.45 | 13.09±1.09 | 4.35±0.19 | 0.00±0.00 | 2.31±0.22 | 8.64±0.78 |
| | 24.56 | α-Cadinene | 4.99±0.56 | 9.06±1.06 | nd | nd | 7.79±0.61 | 8.46±0.58 | 8.32±0.26 | 7.51±0.46 | 11.85±0.34 | 6.88±0.46 | 5.81±0.36 | nd |
| | 24.93 | (E)-Nerolidol | 0.00±0.00 | 5.55±0.33 | 5.08±0.58 | 5.38±0.25 | 7.10±0.35 | 4.10±0.45 | 4.84±0.22 | 3.23±0.13 | 4.71±0.66 | 7.26±0.34 | 4.62±0.39 | 5.69±0.56 |
| | 25.11 | Caryophyllene oxide | nd | nd | 4.60±0.93 | 4.23±0.15 | nd | 4.82±0.32 | nd | 2.96±0.03 | 6.25±0.28 | nd | 3.65±0.58 | 4.45±0.58 |
| | 25.55 | Germacrene B | 16.81±2.56 | 3.42±0.37 | 6.81±1.44 | 5.67±0.44 | 6.57±1.15 | 3.82±0.10 | 4.06±0.23 | 7.22±0.67 | nd | 12.77±2.26 | 30.57±5.11 | nd |
| | 26.49 | Cubebol | nd | nd | nd | nd | 6.06±0.27 | 7.42±0.07 | nd | nd | 7.97±0.53 | 8.21±0.28 | 5.03±0.36 | nd |
| | 27.05 | α-Cadinol | 11.52±5.24 | 12.63±4.13 | 42.44±10.40 | 16.45±3.10 | nd | nd | 8.36±2.12 | 4.65±1.31 | nd | nd | nd | 33.50±7.36 |
| | | Subtotal | 100 | 100 | 100 | 100 | 100 | 100 | 100 | 100 | 100 | 100 | 100 | 100 |

F A

| | | | | | | | | | | | | | |
|---|---|---|---|---|---|---|---|---|---|---|---|---|---|
| 20.44 | cis-3-hexenyl hexanoate | 23.99±1.01 | 18.42±0.71 | 17.92±2.64 | 16.86±2.45 | 19.54±1.10 | 22.70±0.51 | 18.77 | 15.77±0.75 | 22.95±1.40 | 28.51±0.96 | 22.68±1.13 | 0.00±0.00 |
| 21.05 | cis-Jasmone | 59.77±3.00 | 66.83±1.34 | 62.03±1.54 | 61.17±4.79 | 80.46±1.10 | 77.30±0.51 | 60.66 | 72.05±0.81 | 77.05±1.40 | 71.49±0.96 | 77.32±1.13 | 0.00±0.00 |
| 33.41 | Hexadecanoic acid, methyl ester | 16.24±2.04 | 14.75±1.98 | 20.05±3.90 | 21.97±2.42 | nd | nd | 20.58 | 12.18±0.93 | nd | nd | nd | 100.00±0.00 |
| | subtotal | 100 | 100 | 100 | 100 | 100 | 100 | 100 | 100 | 100 | 100 | 100 | 100 |

Data were expressed as mean ± standard error (n = 4). nd, not detected.
