# Supplementary material for: Metabolomic Profiling in Combination with Data Association Analysis Provide Insights about Potential Metabolic Regulation Networks among Non-Volatile and Volatile Metabolites in Camellia sinensis cv Baijiguan
Source: Plants (Basel). 2022 Sep 28;11(19):2557. doi: 10.3390/plants11192557 (PMC9572950; doi:10.3390/plants11192557)
Supplement: Supplementary file 1 [file plants-11-02557-s001.zip › plants-1866919-supplementary/Supplementary material/Supplementary Figures.pdf]

Supplementary Figures

Metabolomic Profiling in Combination with Data Association Analysis Provide Insights about Potential Metabolic Regulation Networks among Non-Volatile and Volatile Metabolites in *Camellia sinensis cv Baijiguan*

Mingjie Chen [1,2,*], Xiangrui Kong [2,*], Yi Zhang [3,*], Shiya Wang [1], Huiwen Zhou [1], Dongsheng Fang [1], Wenjie Yue [4], Changsong Chen [2,†]

[1] College of Life Sciences, Henan Provincial Key Laboratory of Tea Plant Biology, Xinyang Normal University, Xinyang 464000, China
[2] Tea Research Institute, Fujian Academy of Agricultural Sciences, Fuzhou 350012, Fujian, China
[3] School of Life Sciences, Nanchang University, Nanchang 330031, Jiangxi, China
[4] Jinshan College, Fujian Agriculture and Forestry University, Fuzhou, Fujian 350002, China

[†] Correspondence: Changsong Chen (ccs6536597@163.com)

*Equal contribution to this work.

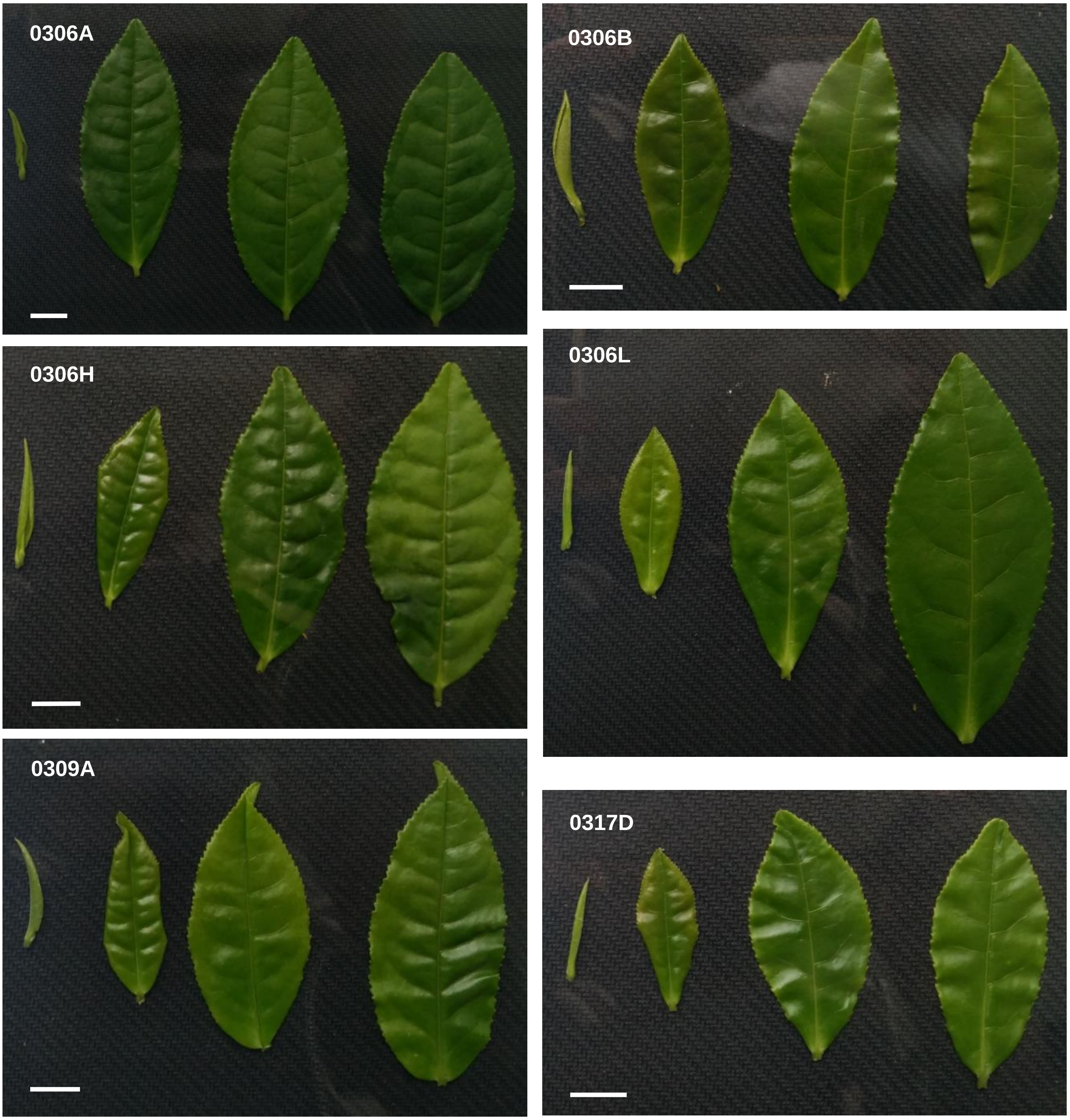

Figure S1. One bud and three leaves of the green leaf half-sibs descendent from *Camellia sinensisi cv Baijiguan*. Bar = 1 cm.

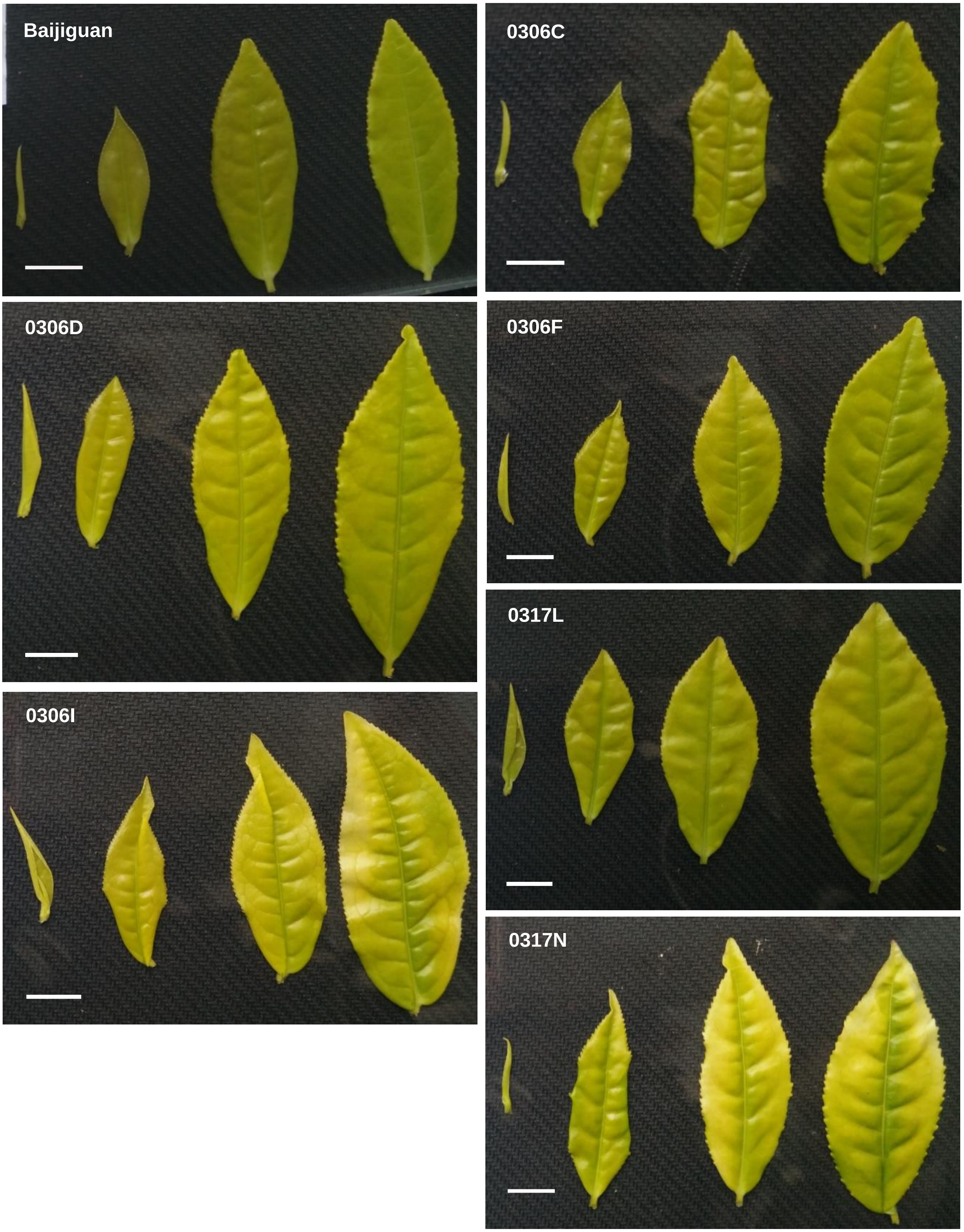

Figure S2. One bud and three leaves of the albino half sibs descendent from *Camellia sinensisi cv Baijiguan*. Bar = 1 cm.

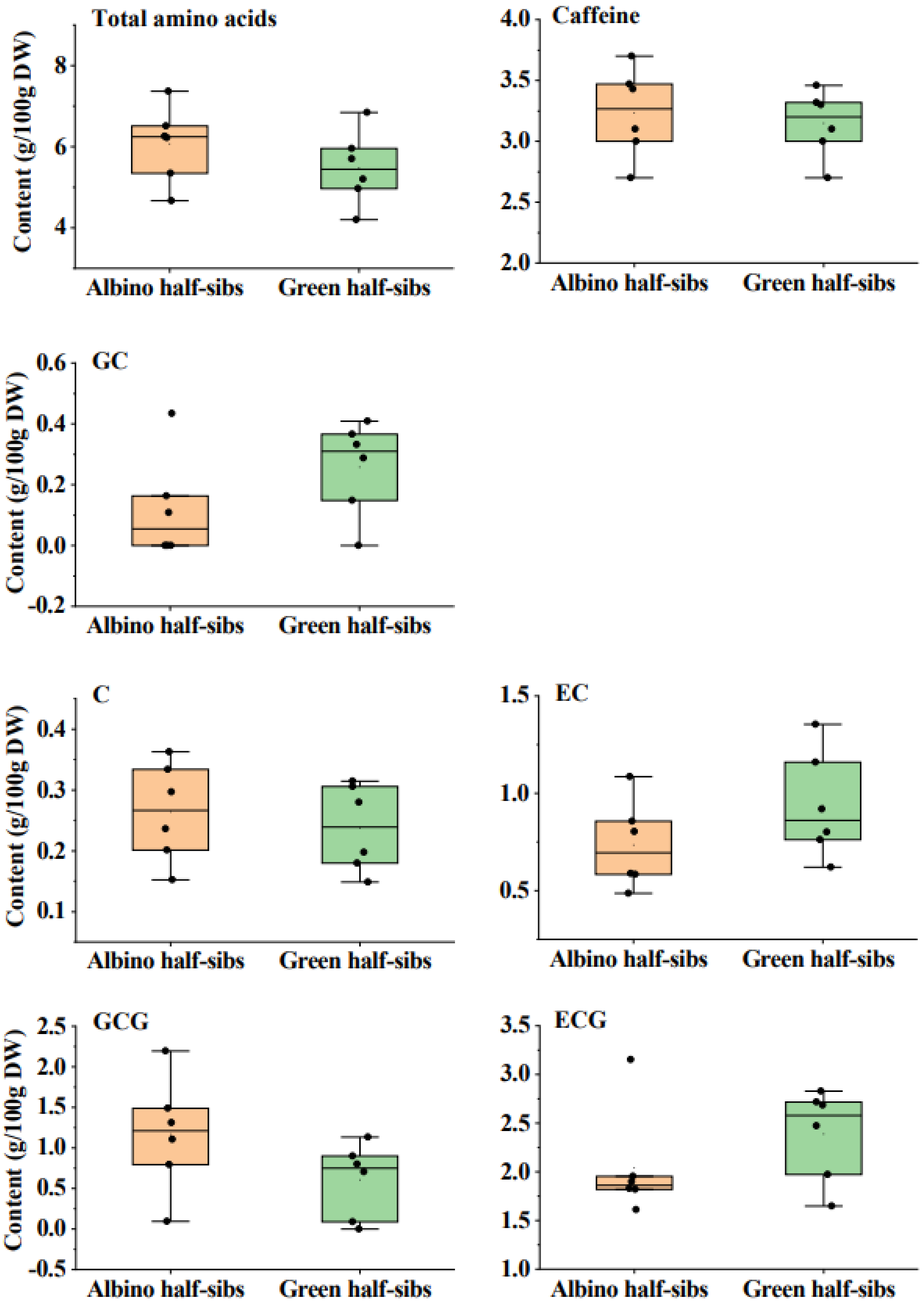

Figure S3. The contents of total amino acids, caffeine, and catechins between the albino half-sibs and the green half-sibs

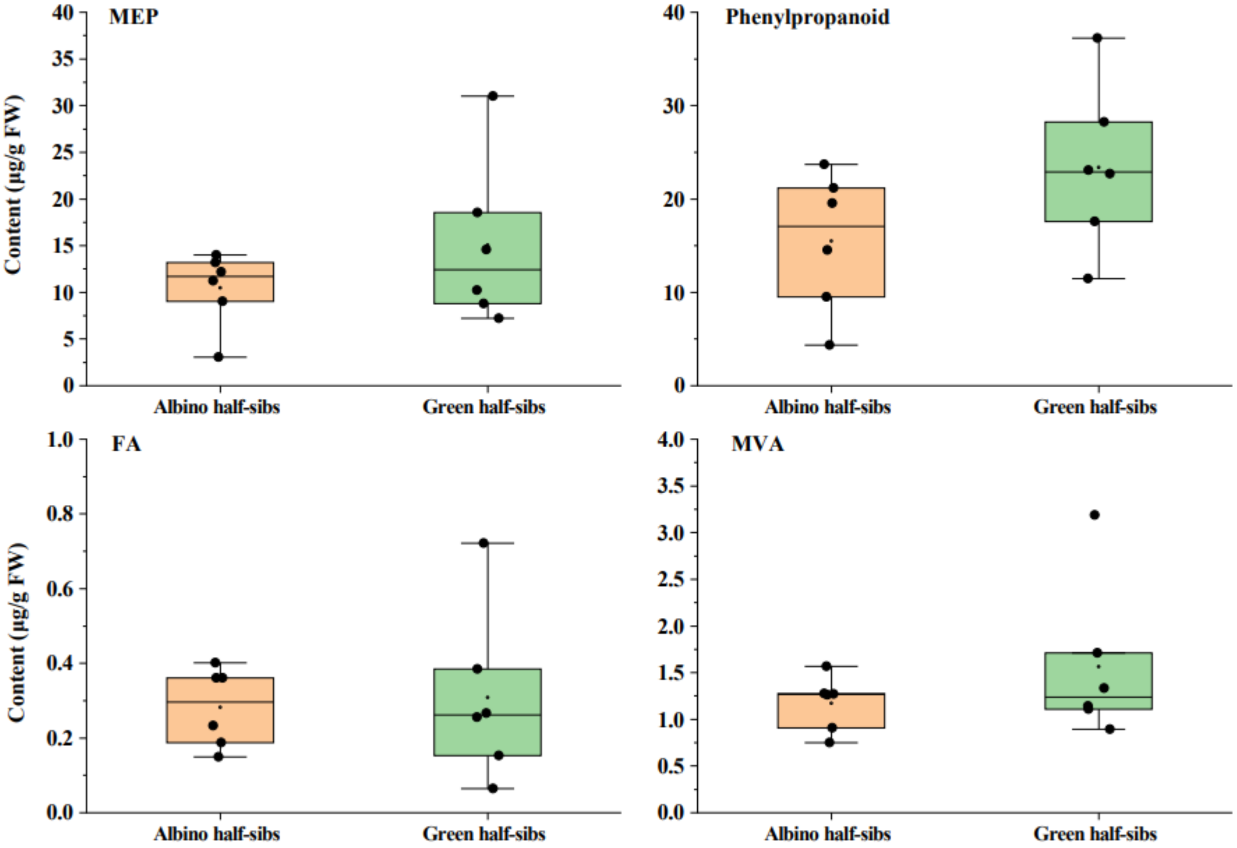

Figure S4. The total volatile contents of individual pathway between the albino half-sibs and the green half-sibs

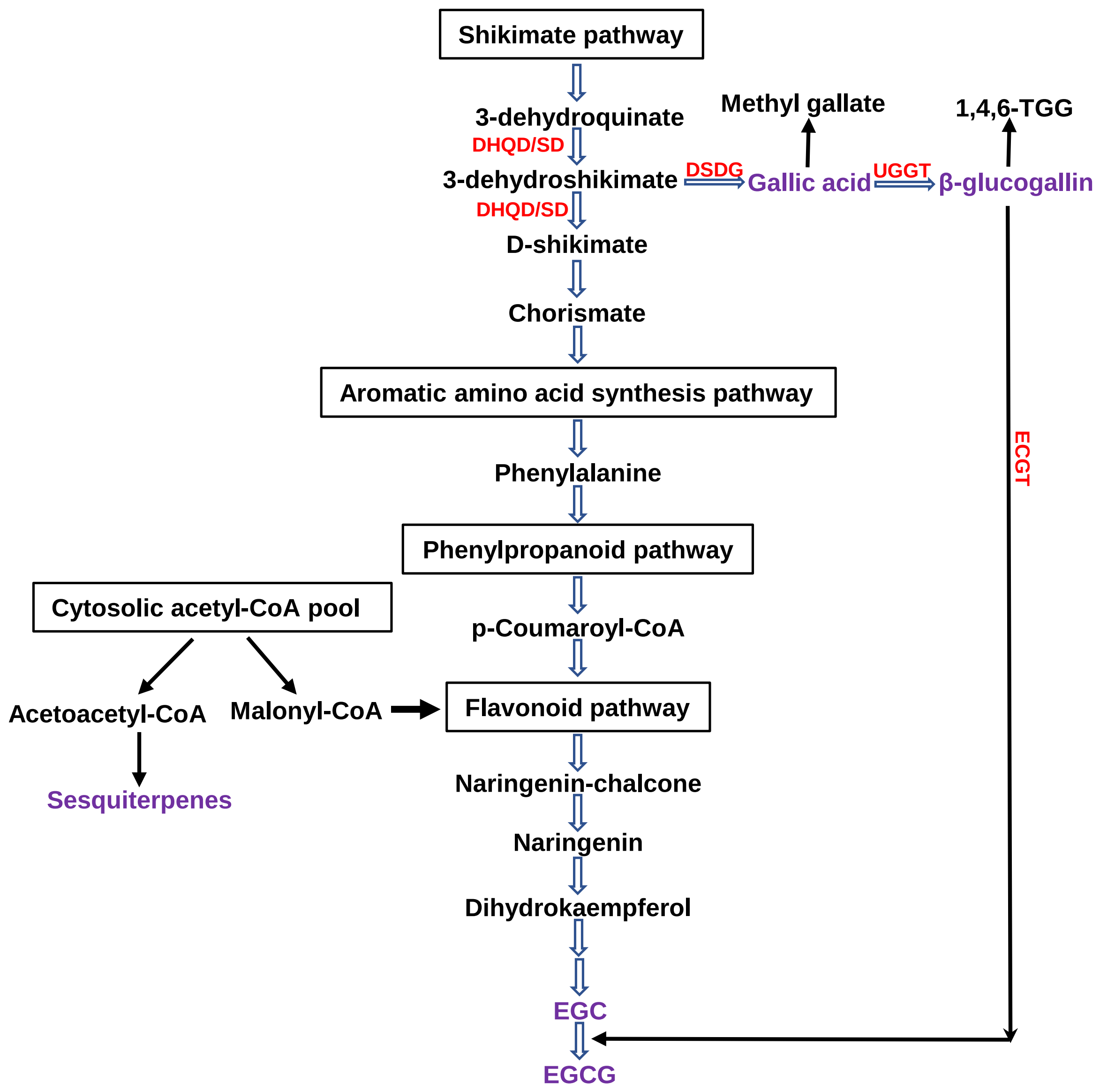

Figure S5. A simplified diagram of catechin synthesis pathway from shikimate. DHQD/SD, 3-dehydroquinate dehydratase/shikimate dehydrogenase; DSDG, dehydroshikimate dehydro-genase; UGGT, UDP-glucose:galloyl-1-O-β-D-glucosyl-transferase; ECGT, epicate-chin:1-O-galloyl-β-D-glucose O-galloyltransferase; EGC, (−)-epicatechin gallate; EGCG, (−)-epigallocatechin gallate;

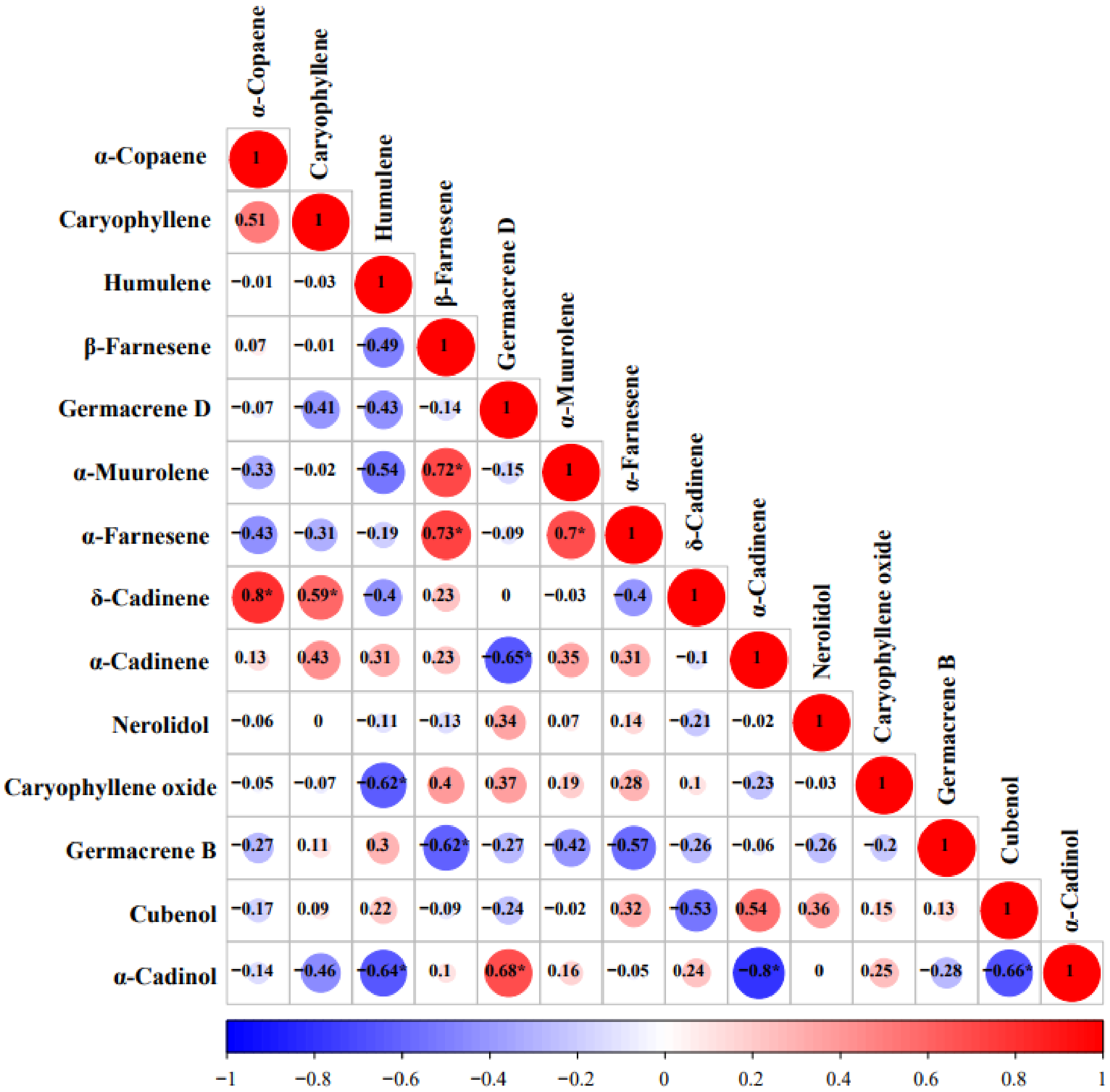

Figure S6. The correlations of volatiles derived from MVA pathway

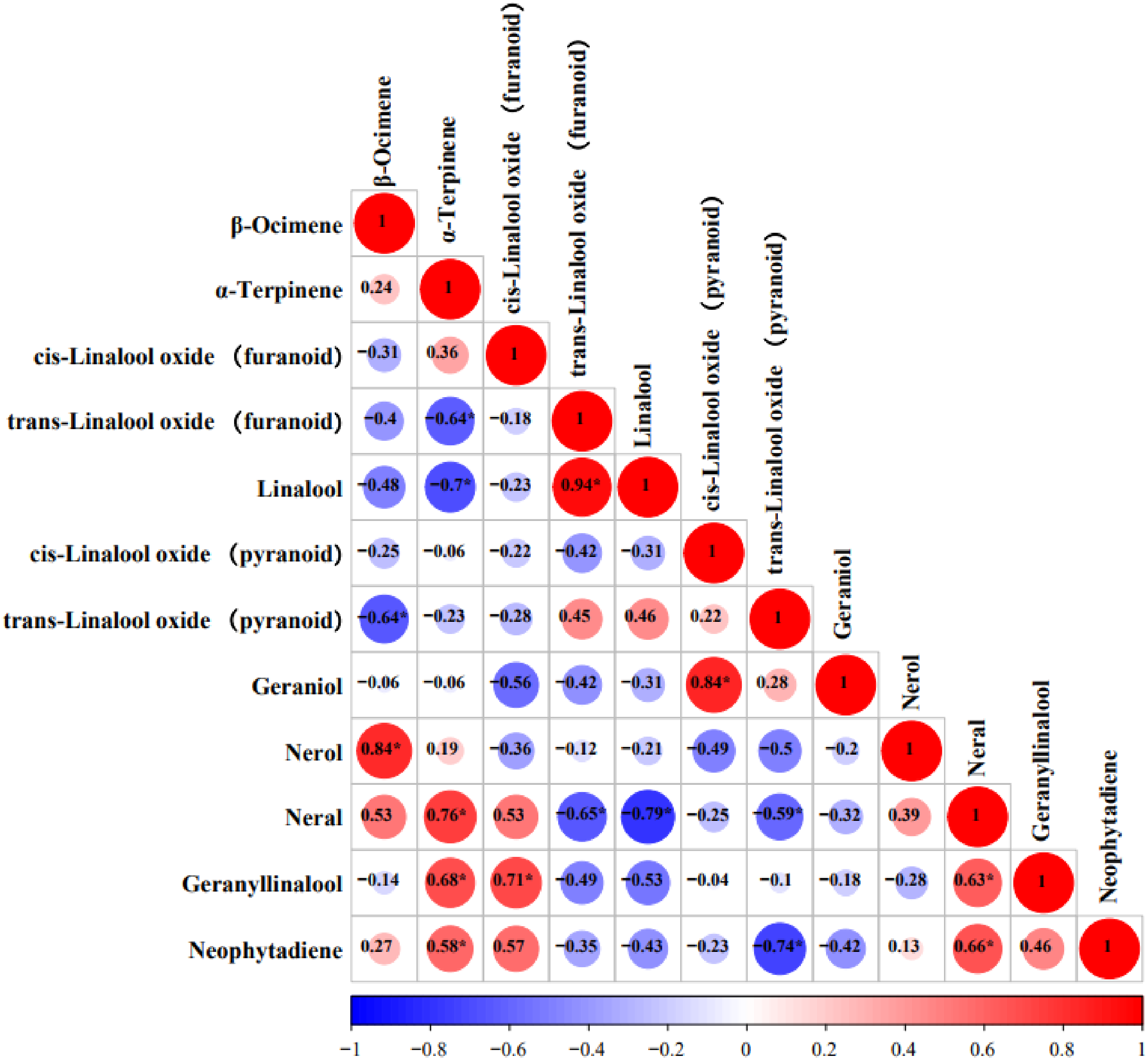

Figure S7. The correlations of volatiles derived from MEP pathway

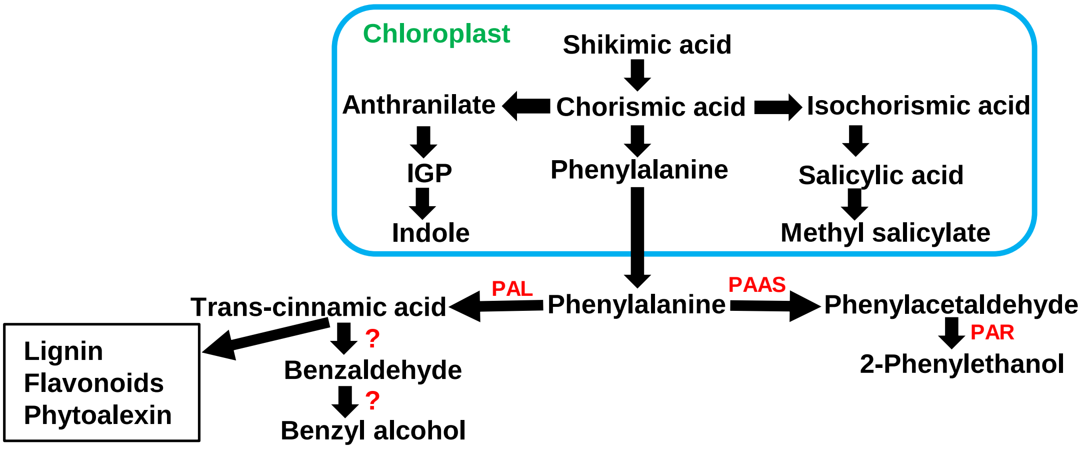

Figure S8. A simplified diagram presentation of shikimate–phenylpropanoid–benzenoid volatile synthesis pathways. PALs, phenylalanine ammonia lyases; PAAS, phenylacetaldehyde synthase; PAR, phenylacetaldehyde reductase
